# Section 4. What the Guidelines Have Missed: A General Discussion of the Faculty: *Highlights of the Asthma Summit 2009: Beyond the Guidelines*

**DOI:** 10.1097/WOX.0b013e3181cb90d9

**Published:** 2010-02-15

**Authors:** Judith R Farrar, Michael A Kaliner

**Affiliations:** 1 LifeSciences Press, Washington DC, USA; Institute for Asthma and Allergy, Chevy Chase, Maryland, USA, and George Washington University School of Medicine, Washington DC, USA

**Keywords:** asthma, guidelines, Expert Panel Report 3, GINA, Asthma Summit

## Abstract

The sessions during the 2 days of the Asthma Summit focused largely on some specific aspects of the current European and U.S. guidelines for managing asthma. By way of summary, the faculty addressed the question of what they thought the guidelines missed, starting with consideration of those aspects of their own practice management that they believed are not clearly discussed in current guidelines.

## Asthma summit 2009: what the guidelines have missed, a general discussion of the faculty

### Dr. Calhoun

For me, an issue is that the guidelines don't discuss the concept of personalized therapy--the notion of individual phenotypes based on genetic, genomic, functional genomic, or proteomic or even clinical demographic predictors. Do the rest of you think that personalized medicine is important, or is it simply the newest catch phrase?

### Dr. Busse

I think this was addressed in some of the later sections of the Expert Panel Report 3 (EPR-3) and also in an accompanying editorial in *Journal of Allergy*. Personalized medicine is emerging, and we've talked about it here with regard to phenotypes--including even different phenotypes for exercise-provoked bronchospasm. The unifying idea is that by looking at specific patterns of symptoms and then patterns of responses, we'll have a far more educated guess as to the best therapeutic approach. We're not yet ready for genotyping, which is a far more advanced approach that may be available down the road.

### Dr. Luskin

I think we need to take that with caution because personalized medicine depends upon who's practicing it. One of the reasons the Guidelines Committee was set up in the first place in 1989 was because there was so much variability of practice, and the variability was associated with poor outcomes and high cost. So while I, like any one of you in this room, would like to be practicing personalized, patient-centered medicine, I'm not sure that I can say the same for those taking care of the majority of "standard asthmatics" in this country.

### Dr. Hargreave

As you say, it is a question of who is doing the treating. But, as specialists, we need to be above this. Why are we specialists if we can't do this better than everybody else? So, in terms of future guidelines, I think it might be advantageous to have some separation between the specialties.

### Dr. Busse

The National Asthma Education and Prevention Program (NAEPP) tried to tactfully address this in the guidelines in terms of the criteria for referral to an asthma specialist. But as discussed these past 2 days, despite these guidelines, control is not achieved in the majority of asthma patients, particularly those with more severe disease. Anything we can do to improve it is a good idea.

### Dr. Kaliner

Consider this case. A patient comes in to my office with moderate symptoms and an forced expiratory volume in one second (FEV_1_) of 45%. What do I do?

### Dr. Chipps

First, see how much they reverse.

### Dr. Kaliner

No reversibility; standard bronchodilator.

### Dr. Chipps

Do a chest x-ray; take their smoking history.

### Dr. Kaliner

No smoking; chest x-ray is normal.

### Dr. Chipps

What's their *α*1-anti-tryptase level and phenotype?

### Dr. Kaliner

Normal and negative.

### Dr. Oppenheimer

Does this person have normal lung volumes or just normal spirometry?

### Dr. Kaliner

Everything is commensurately reduced, so the FEV_1 _is down 45%. Small airways are down, and the patient has some shortness of breath.

### Dr. Chipps

Is the ratio normal?

### Dr. Kaliner

The ratio is about 75%.

### Dr. Luskin

You're describing a patient whom we all see, who evidences a significant dichotomy between the physiologic, pathologic, and clinical aspects of disease. I would treat the patient. If I'm convinced that there's no response to beta-agonists, I might give a short trial of prednisone.

### Dr. Kaliner

What do you tell the patient? The patient who is, say, 40 years old says, "Am I going to die of shortness of breath?"

### Dr. Luskin

You watch, and you do what we are taught by the guidelines to do in terms of that risk of progressive loss of lung function that maybe we can't do anything about.

### Dr. Colice

I think this is really an important question because within the guidelines now there are the 2 different risk domains; and one of the risk domains is for accelerated decline in lung function. Presumably, this patient with asthma has had an accelerated decline in lung function over time, so I think vigorous treatment would be required. I would put them on prednisone for a while and probably a small particle inhaled corticosteroid to see if I can get some small airway improvement.

### Dr. Busse

Unfortunately, I agree that we see this patient fairly often, and it can be confusing because there is both an obstructed and a restricted pattern (hyperinflation). I agree with the suggested course of corticosteroids. If that doesn't help, then we may want to look at the differential diagnosis of obstructive airway disease. Some studies have indicated that, in very severe asthma, there can be airway trapping and airway parenchymal uncoupling, with a high buildup of residual volume leading to restrictive and obstructive components. Unfortunately, this type of patient doesn't always respond very well to our current therapies.

### Dr. Calhoun

Another lesson from this case is that establishing good relationships between the pulmonary and the allergy communities is critically important.

### Dr. Colice

Agreed. There are other diagnoses to consider, and in this case complete pulmonary function tests and computed tomography scans during inspiration and expiration could be helpful.

### Dr. Oppenheimer

Earlier, Dr. Busse mentioned referral. According to the guidelines, referral is at step 3 or step 4, depending upon the patient's age, yet we know that the majority of patients and doctors in this country do not recognize the severity of the disease. So are a lot of people being missed because the primary care doctor isn't acknowledging that asthma is significant?

### Dr. Busse

I think to a certain extent that is true. However, hospitalization rates have dropped off, so I think that more people are getting better care. I think the criteria we need to look at now are how frequently patients are using prednisone bursts. Once or twice a year is acceptable. I also think some of the questionnaires, like the Asthma Control Test (ACT), can be helpful. What we need to do now is to educate the patients. A good example is cholesterol treatment. Virtually everybody wants to know what their cholesterol is because they know it's a big risk factor for heart disease.

### Dr. Oppenheimer

So maybe the next set of guidelines can talk about an ACT score. I would also argue for beta-agonist use, if we can get the pharmacists involved, and then prednisone, which may be a better mechanism for referral.

### Dr. Busse

Absolutely.

### Dr. Bukstein

Let's get back to what's missing in the guidelines. As mentioned earlier, I think one thing that's missing from asthma guidelines that is in most other chronic disease guidelines is the role of lifestyle change.

### Dr. Colice

Agreed.

### Dr. Spector

Another item missing might be updated guidance on new triggers. For example, anti-mouse antibodies have now been associated with increased asthma symptoms, especially in the inner city. Presently, I don't do mouse skin tests. Should I? Is this something we should be recommending in future guidelines?

### Dr. Busse

I think that the specific risk factors depend upon where you live. If you live in the inner city, there's a huge load of cockroach antigen, and it's complicated by the presence of mouse dander and fecal material. There also may be some protectivity--the data that dogs can be protective but not cats are interesting. Is there endotoxin or fecal material attached to the dog, which then has an effect on the airway response? I don't think we have explored the environment as much as we should. There's also the area of microbiota and the influence of intestinal bacteria. For example, clostridium is associated with a shift toward T_H_1 and lactobacillus toward T_H_2. These are some very new and exciting areas.

### Dr. Spector

The other thing missing is the role of diet, which has been talked about only peripherally. There are some interesting data about omega-3's and fish oil blocking exercise-induced bronchoconstriction in various models. Vitamin D and folic acid deficiency have been incriminated in asthma. Should diet be covered better in the future? Should we be advocating specific diets for our patients?

### Dr. Calhoun

The panel attempted to be completely evidence-based, and so the recommendations were driven by the best evidence gathered. While there are some intriguing things about diet and other factors, it probably didn't rise to the level of being incorporated into the guidelines.

### Dr. Luskin

We also need to understand that people who are IgE makers are capable of making IgE to things that we don't usually think about--like Miller moths and Japanese beetles. In terms of diet, there is intriguing information about vitamin C, omega-3's, and vitamin D. But are these ready for guideline primetime? I don't think so. The lifestyle issues that Dr. Bukstein raised include learning how to eat healthier and decreasing body mass; and there's a substantial body of evidence that these lifestyle approaches will dramatically reduce the patient's pharmaceutical burden and improve quality of life.

### Dr. Kaliner

What about allergy immunotherapy? Where does that fit in?

### Dr. Colice

First, let me say that I think guidelines are great because they help us not only to crystallize our thinking but also to point out areas where we don't know enough about clinical practice. That's one aspect of guidelines that has not been emphasized. Any time you see anything less than evidence level A or B, it tells you that we need more information, more research.

With regard to immunotherapy, frankly, I do not recommend it for my patients with asthma unless they have a big rhinitis component.

### Dr. Kaliner

In our own practice, we use limited immunotherapy for allergic rhinitis because it's easy enough to manage that disease unless it's very severe, which is not common. However, we use immunotherapy for our allergic asthmatics at step 1. We use it as a first time indication. Our belief, on the basis of the outcomes we see, is that in many patients successful immunotherapy significantly reduces (even eliminates) the asthma.

### Dr. Luskin

Would you also use immunotherapy for patients with rhinitis and bronchial hyperreactivity, but no asthma? These people have been shown to be an at-risk subset for developing asthma.

### Dr. Kaliner

I'm not sure that I see that in practice, but I might.

### Dr. Chipps

I agree with Dr. Luskin. I think the patient with rhinitis who is beginning to have some exercise-related "asthma-like" symptoms is the perfect patient to treat.

### Dr. Busse

In the U.S. guidelines, immunotherapy was placed for consideration between steps 2 and 4; it's not advocated in the GINA guidelines. The data exist, but the studies are not as clear as for medications. Another consideration is whether parenteral immunotherapy is really what we want to do. I'm not sure where sublingual immunotherapy will fit, but some of the data suggest an effect on the upper and lower airways. But doing the studies and getting type A evidence is going to be very difficult, requiring long-term studies.

### Dr. Kaliner

This is what the guidelines are missing. As a consequence, a sizable portion of the asthma-treating community, without guidelines, believe that immunotherapy has no role in managing allergic asthma. So many patients aren't getting allergy assessment or treatment even though they might benefit from it. I think this is a significant hole.

### Dr. Colice

I agree. There's a lot of extraordinarily good information in the EPR-3, but I came away without a sense that immunotherapy was beneficial in asthma. As a consequence, I do not prescribe it.

### Dr. Luskin

So, to summarize, the NAEPP Guideline Committee felt that it was more important to be evidence-based than to make a statement based on incomplete evidence--even if that incomplete evidence was guiding current practice.

### Dr. Busse

Yes. We were cautious.

### Dr. Storms

Dr. Busse, do you think for the next rendition of the guidelines there will be enough data from Europe to support recommendations for sublingual or oral dissolvable immunotherapy for asthma?

### Dr. Busse

It's a good question, and I don't know the answer.

### Dr. Chipps

I am concerned about the seasonality of viral infections as a risk domain that drives a lot of patients in terms of their morbidity and that is not specifically discussed with regard to not stepping down treatment.

### Dr. Busse

As noted earlier, stepping down is not addressed very well. In this regard, I think an area that is particularly intriguing is the "September epidemic" in children. During the summer months, many children stop their medications because they are not in school, and they sometimes feel better. Then they go back to school and "boom." We thought it was probably due to rhinovirus, but in a study we did one of the major risk factors was cockroach sensitivity. That raises the question again about the interplay between environmental factors, viruses, and antigens. A major risk factor for having an asthma exacerbation is being allergic, and how this interplays with viral infection is very intriguing.

### Dr. Hargreave

I agree that it needs to be taken into consideration. You showed that virus infection increased the late response to allergen inhalation.

### Dr. Busse

Yes, almost 15 years ago. The interaction with virus changed things a lot.

### Dr. Hargreave

Anecdotally, we've seen that if a patient has eosinophilia and gets an infection on top of it, that patient is much more likely to have symptoms.

### Dr. Busse

We really haven't talked about the susceptibility to events that are occurring because these are not things that we measure. I think we need to look at different outcomes depending upon the stimulus. Until we do that, it's not easy to show effectiveness, remembering that effectiveness is not just an improvement in FEV_1_.

### Dr. Calhoun

So I'd like to bring this back to a question that was raised at the very beginning of the symposium, in the opening debate between Dr. Busse and Dr. Bousquet. Several folks articulated that we really need to have a universal guideline for asthma. I'd like a little discussion on that point, recognizing that there are cultural differences. For example, in Japan, the use of inhaled drugs is not very well accepted. There are also important differences in the regulatory landscapes between countries. So, is the concept of a universal guideline for asthma something that's interesting but unachievable or is there a way forward that will accommodate some of these regulatory and cultural differences?

### Dr. Busse

If you look at guidelines from different countries, there are many components that are in agreement: the characteristics of the disease and the steps of severity. Then the countries go to their local environments as to how to do things.

### Dr. Colice

It's an interesting question. You know, of course, in chronic obstructive pulmonary disease (COPD) there's a much clearer agreement on international guidelines with the Global initiative for chronic Obstructive Lung Disease (GOLD) approach, so I think there is a possibility of achieving that. But I think we are a lot better off than we were 10 years ago. There's a lot more clarity as to where we should be going.

### Dr. Luskin

That brings up another issue, which is what needs to happen to optimize the effectiveness of the guidelines and how to put them into operation. They are a fantastic reference, but in practical terms not particularly useful, especially for the group that sees the most asthmatic patients. The Guideline Implementation Committee suggested 22 key messages and then 6 "really" key messages. It might be worthwhile to look at what National Committee for Quality Assurance (NCQA) and the American Medical Association (AMA) conjoint panel suggested as useful for judging quality in asthma care. They suggested the following: (1) there needs to be assessment of control at every visit, and the ACT is an easy way of doing that; (2) there needs to be assessment of risk at every visit; (3) every patient should get a written action plan; (4) appropriate pharmacotherapy should be controller medication for persistent asthma, and they favor looking at a controller-to-reliever ratio to predict patients at high risk for exacerbations; (5) smoking cessation is critical; (6) follow-up after an urgent visit should happen within 1 to 2 weeks, and inhaled corticosteroid should be prescribed at discharge from urgent care. According to the NCQA and the AMA, those are what would most likely help people implement the guidelines.

### Dr. Colice

I would implement the guidelines in 2 different ways. First, we should implement in a uniform way the grade A evidence. Evidence below that should be prioritized for research, which is where the NHLBI comes in.

### Dr. Hargreave

In summary, asthma is a disorder defined by clinical, physiological, and/or pathologic criteria. But basically we see asthma as variable airflow obstruction. That's how we make the diagnosis. That's what we're talking about when we talk about asthma. So the guidelines definitions are descriptive. There's no primary defining characteristic. They don't tell us that when we talk about asthma, we're talking about airflow limitation, which varies over short periods of time; they don't really tell us how to make the diagnosis, though there is explanation for how that can be done. However, they imply that there are several characteristics that you have to have to have asthma; but with all this heterogeneity going on, that isn't the case. So we are confused. We've talked about heterogeneity. There's heterogeneity among the components in people with airway disease, so they can have 1 or several of these different abnormal characteristics.

An editorial in the *Lancet *about 2 years ago suggested that we should abandon asthma as a disease concept. I agree with that. Continue to use the term as an abnormality of function and exactly describe each subject using measurements. We need to assess what the different things are that are contributing to each patient's symptoms, and then we treat them. This is how we can personalize treatment; and that's what we've been talking about, in fact, throughout this meeting.

## Note

Sponsored by LifeSciences Press, LLC, and supported by an unrestricted educational grant from Sepracor, Inc. All content has been derived from the Asthma Summit 2009, which was sponsored by the SRxA Institute for Professional Education and Medical Education Resources and funded by unrestricted educational grants from Sepracor, Inc., Abbot Laboratories, Graceway Pharmaceuticals, Strategic BioSciences, and the TREAT Foundation

